# Development and Validation of Computational Fluid Dynamics Models for Prediction of Heat Transfer and Thermal Microenvironments of Corals

**DOI:** 10.1371/journal.pone.0037842

**Published:** 2012-06-11

**Authors:** Robert H. Ong, Andrew J. C. King, Benjamin J. Mullins, Timothy F. Cooper, M. Julian Caley

**Affiliations:** 1 Fluid Dynamics Research Group, Curtin University, Perth, Western Australia, Australia; 2 Atmospheric Environment Research Centre, Griffith University, Brisbane, Queensland, Australia; 3 Curtin Health Innovation Research Institute, Curtin University, Perth, Western Australia, Australia; 4 Australian Institute of Marine Science, UWA Oceans Institute, Perth, Western Australia, Australia; 5 Australian Institute of Marine Science, Townsville, Queensland, Australia; Leibniz Center for Tropical Marine Ecology, Germany

## Abstract

We present Computational Fluid Dynamics (CFD) models of the coupled dynamics of water flow, heat transfer and irradiance in and around corals to predict temperatures experienced by corals. These models were validated against controlled laboratory experiments, under constant and transient irradiance, for hemispherical and branching corals. Our CFD models agree very well with experimental studies. A linear relationship between irradiance and coral surface warming was evident in both the simulation and experimental result agreeing with heat transfer theory. However, CFD models for the steady state simulation produced a better fit to the linear relationship than the experimental data, likely due to experimental error in the empirical measurements. The consistency of our modelling results with experimental observations demonstrates the applicability of CFD simulations, such as the models developed here, to coral bleaching studies. A study of the influence of coral skeletal porosity and skeletal bulk density on surface warming was also undertaken, demonstrating boundary layer behaviour, and interstitial flow magnitude and temperature profiles in coral cross sections. Our models compliment recent studies showing systematic changes in these parameters in some coral colonies and have utility in the prediction of coral bleaching.

## Introduction

An increase in the magnitude and frequency of stress-induced coral bleaching in the past two decades is likely due to a variety of stressors [1]. The most common cause of coral bleaching is an elevation of sea surface temperature (SST) combined with elevated solar irradiance [2]–[4]. Because corals thrive close to their upper thermal tolerance threshold [5], bleaching is expected in response to a 1–2°C temperature increase over a prolonged period. Some coral species, however, bleach more readily than others [3], [6]. While bleaching can be strongly correlated with SST, several experimental studies have also shown a clear difference between coral surface (tissue) temperature and SST [7], [8]. This temperature divergence is likely due to the physics of heat transfer and fluid flow, coupled with other interacting phenomena, such as the influence of coral porosity and permeability, as well as differences in the structure and growth forms of different coral species. Here, we begin to explore the effects of these coupled processes using a computational fluid dynamics framework with a view to providing a better understanding of the role these parameters play in coral warming and resultant bleaching.

The calcium carbonate skeleton of corals is predominantly composed of the mineral’s aragonite polymorph, which has a density of 2.94 g cm^−3^ [9], [10]. The highly porous structure and permeability of coral skeletons, and the morphologies of their colonies, may play a significant role in determining coral surface temperatures. In spite of their potential importance, the influence of coral porosity and permeability and colony shape on coral thermal microenvironments and their roles in determining the susceptibility of corals to bleaching is yet to be properly addressed. Recent suggestions of changes in growth rates of massive and branching corals on the Great Barrier Reef [10], [11] and West Australian Reefs [12] would indicate potential changes in bleaching susceptibility should these mechanisms prove to be important. Furthermore, the growth of coral reefs is highly dependent on the framework provided by corals and its degradation by physical, chemical and biological processes [13]. While bioerosion, predation, sedimentation and hurricanes can all reduce coral growth by damaging coral tissues, they may also affect any relationship between fluid dynamics and heat transfer, and consequently, the susceptibility of corals to bleaching. For example, the bioerosion of corals through boring, etching and grazing organisms, will lead to increased (local) skeletal porosity [13], [14].

The mechanisms that underpin coral bleaching remain unclear, due in part, to the difficulty of obtaining accurate measurements and predictions from in-situ monitoring of the complex environments experienced by corals in both time and space [1]. Meanwhile, laboratory studies can be confounded by the susceptibility of most coral species to handling stress, and the difficulty in precisely imitating field conditions [1]. Moreover, conventional laboratory methods are often limited for determining values of many parameters of interest within the interior of corals. These parameters (i.e., flow, pressure, temperature, etc), related to coral morphology, are likely to be important determinants of mass and heat transfer in corals, and ultimately may be important determinants of their sensitivity to bleaching [15], [16].

In contrast to experimental techniques, numerical modelling methods allow for detailed interrogation of these parameters without difficulty, thanks to the availability of commodity computing resources. For example, computational fluid dynamics (CFD) is a powerful tool with which to investigate systems involving fluid flow, heat transfer, and associated phenomena by means of computer-based numerical simulation [17]. A CFD study can be divided into three-steps: pre-processing, computation, and post-processing. First a geometric model is created, which is then broken down into small finite volumes (termed volumetric cells). Physical properties and operating conditions of the model are then specified in a solver, which uses efficient algorithms to solve a system of simultaneous equations. The solver is then used to solve these equations governing the flow and heat transfer for a wide spectrum of possible environmental conditions. Post-processing is then used to analyse and extract the required results from the flow field solutions. Further advantages that CFD has over experimental approaches include low cost, speed, and the ability to simulate both realistic and ideal conditions [18]. However, as with all models and simulations, CFD solvers must be validated.

Here we report on the development of CFD models and numerical solvers for steady state and transient simulations of coral microenvironments. These models couple the effects of fluid flow, coral porosity and permeability, spectral irradiance, and the heat transfer in and around hemispherical and branching corals. We then validate these models using a published case study [8]. The aim of the present work is to develop and test the validity of a series of CFD models applicable to the study of coral bleaching, at least under laboratory scale conditions (laminar flow and overhead (90° incident angle) radiance). However, with further development and validation, these CFD models can also be used to investigate the influence of a broader spectrum of environmental parameters on coral tissue warming. Such parameters could include flow variations (e.g. turbulent flow), emissivity or absorptivity of the coral surface, sea-state (e.g. waves), and cloud cover. Other well-known stressors that cause bleaching, can also be investigated using CFD simulation, such as cold-water bleaching [19]–[21], increased or decreased salinity [22]–[24], and sedimentation and turbidity [25], [26]. An overview of the computational framework used for a subset of environmental parameters is presented here. A more complete treatment of a spectrum of parameters will be presented elsewhere.

### Flow and Thermal Regions in Corals

**Figure 1:**
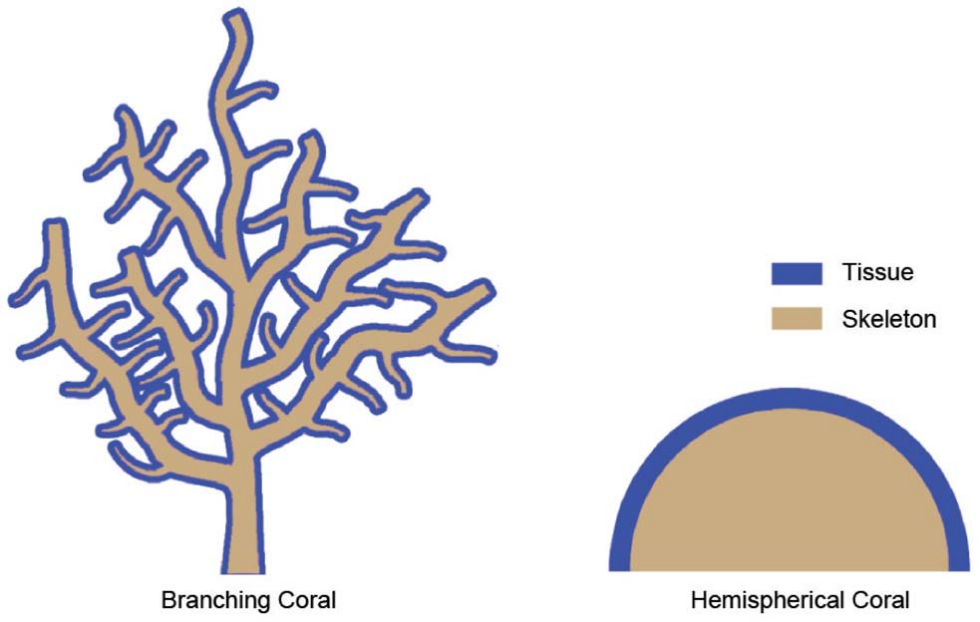
The distribution of living tissue and skeletal matrix in branching and massive corals.

The living tissue of corals that connect adjacent polyps forms a thin layer over the outermost few millimeters of its calcareous skeleton (Figure 1) [27], [28]. The flow of water through the coral tissue and skeleton (if it occurs), should significantly influence the thermal microenvironment of the coral. To our knowledge, there are no published reports of flow through living coral tissue or their skeleton. Therefore, in constructing these models, we have developed models that allowed us to evaluate a diverse range of scenarios. To do so, however, necessitated assumptions that will need to be tested in future work. In particular, we assumed that there is unlikely to be any significant permeability through intact living tissue, however, in many cases, the activity of borers and coral grazers will result in openings allowing percolation and increased skeletal porosity. Coral species with denser skeletons experience less percolation. Our validation case study [8] used cut pieces of coral, which would have significant permeability on the cut faces. Thus, in this work we assumed coral tissue had percolation flow equivalent to 5–10% porosity.

Assuming a starting condition whereby the temperature of the coral and the surrounding water are the same, heat transfer within and around corals will be affected by three main mechanisms: (1) the rate of incident radiation absorbed by the exposed tissue surface, (2) the rate of heat loss due to convection from the tissue and skeleton into the surrounding water, and (3) the rate of heat conduction from the tissue into the skeleton. The models we present here consider all of these mechanisms. It is, therefore, necessary to specify two thermal and porous regions, the tissue and skeleton, in our models. Our models also assume thermal effects of mass and nutrient transfer during photosynthesis to be negligible.

### Thermal Physics in Hemispherical and Branching Corals

To aid understanding of heat transfer processes in corals, a simple heat balance between heating and cooling in a coral can be written as
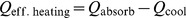
(1)where 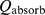 is the heat absorbed by a coral due to solar irradiance and 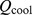 is the energy that is transferred from the coral surface to the surrounding environment. Applying Newton’s law of cooling and rearranging the above equation, 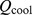 and 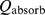 can be expressed as


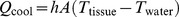
(2)


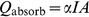
(3)

where *h* is the heat transfer coefficient between the coral surface and the surrounding environment, *I* is the total irradiation, 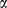 is the tissue absorptivity, and *A* is the surface area of the coral (Table 1). Heat transfer is directly proportional to surface area, whereas heat capacity is proportional to volume. Hence the *A*/*V* ratio influences heat accumulation and dissipation. Branching corals have larger surface area to volume, *S* = *A*/*V*, than massive corals and therefore can be expected to acquire and dissipate more heat.

**Table 1:** List of symbols and their definitions.

*α*	tissue absorptivity	*h*	heat transfer coefficient
*A*	coral surface area	*I*	irradiance
*T_t_*	coral tissue temperature	*T_f_*	ambient fluid temperature
U	superficial velocity vector	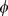	porosity
*v*	kinematic viscosity	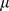	dynamic viscosity
*p_c_*	coral density	*p_f_*	fluid density
*C_pc_*	coral heat capacity	*C_pf_*	fluid heat capacity
*k_c_*	coral thermal conductivity	*k_f_*	fluid thermal conductivity
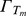	medium thermal diffusivity	*Pr*	Prandtl number

The rate of temperature rise in the coral can be written as
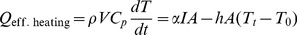
(4)where *p* is the coral density, *C_p_* is the coral specific heat capacity, and 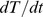 is the rate of temperature rise in the coral. Thus, from equations 2, 3, and 4 the temperature rise in a coral should be highly dependent on its ratio of *A* and *V*. Defining a surface area to volume ratio, *S* = *A*/*V*, the above equations can be solved and a time constant, 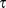, can be identified for the coral, such that
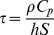
(5)This equation illustrates the strong dependence of the time constant (or heating rate) on *S* (as *S* varies considerably between hemispherical and branching corals). The time constant, 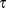, therefore increases with increasing volume and decreases with increasing surface area, and the *A/V* ratio will be an important determinant of the speed with which an equilibrium temperature is reached, as well as the final temperature achieved. This expression also includes a dependence on the boundary layer thickness due to the convective heat transfer coefficient ‘*h*’.

While we recognise that coral can not be completely characterised by ideal geometric shapes, for our purposes here, we define the shapes of massive and branching corals as hemispherical and cylindrical, respectively. While a caricature of nature, this conceptualisation represents the ends of the shape spectrum of corals. For the corals investigated here the surface areas of hemispherical and branching corals are given by 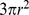 and 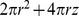, respectively, where *r* is the radius and *z* is height of a cylindrical (or branch) section. The corresponding volumes for the hemispherical and branching corals are 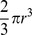 and 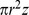, respectively. Recent experimental work [29] has measured thermal heterogeneity on a micro-scale in individual polyps. Our current geometry assumes homogeneity, however, a more spatially resolved geometry could readily be substituted.

## Methods

### Computational Fluid Dynamics

This study was conducted using OpenFOAM (Open Field Operation and Manipulation) [30]. OpenFOAM is an open-source suite of software applications, libraries and utilities for problems involving continuous fields, such as fluid dynamics. The physical laws governing porous media, fluid flow, and heat transfer processes must firstly be defined as differential equations. The mass continuity governing equation was developed from the mass balance over a control volume (cell), fixed in space, through which the fluid flows and can be written as
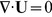
(6)where U is the superficial velocity vector. Because the focus of our model is at the laboratory scale, any differences in seawater density will be very small, and are therefore ignored, and fluid flow is treated as being incompressible. However, the application of this modelling framework at larger scales, will require these assumptions to be re-visited because changes in density are likely with changes in salinity and temperature.

The momentum governing equation for laminar, viscous, incompressible, single-phase water flow through a porous medium is given by
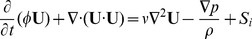
(7)where 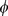 is porosity, *v* is kinematic viscosity, *p* is pressure, and *k* is thermal conductivity (Table 1). The flow sink term, *S_i_* is composed of two parts, a viscous loss term and inertial loss term, creating a pressure drop that is proportional to the velocity and square of the velocity, respectively
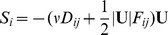
(8)where *D_ij_* and *F_ij_* are represented as the scalars D and F. This is the classical Darcy-Forchheimer equation. In this study, corals were assumed to possess homogeneous permeability and were treated as having isotropic permeability. The Blake-Kozeny equation specifies viscous energy loss primarily in laminar flow as [31]

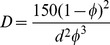
(9)where, *d* is the diameter of the coral sample under investigation. The Burke-Plummer equation denotes the kinetic energy loss primarily in turbulent flow as [31],
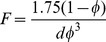
(10)Because the pressure drop for low velocity flow, as modelled here, is predominantly determined by viscous forces, the inertial or kinetic energy term can be omitted. However, for high velocity flow, kinetic forces dominate the pressure drop and must be retained.

The final transport equation is the energy governing equation, which accounts for heat flow within the models, including the effects of incident solar radiation. In our porous zone models, we have treated all the corals as isotropic media with uniform porosity and permeability. Although this may not be realistic in some cases, we consider this to be a reasonable starting approximation. To describe the temperature distribution in the porous coral tissue and skeleton, we assumed an instantaneous local thermal equilibrium whereby *T_c_* = *T_f_* = *T* (where *T_c_* and *T_f_* are the temperatures of the coral and the fluid, respectively). It can be expressed as
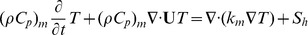
(11)where *S_h_* accounts for heat source (such as the incident radiation on the tissue, 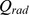). Averaged material properties for the porous media were calculated using



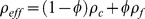
(12)

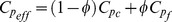
(13)

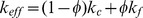
(14)where equations 12, 13, and 14 represent effective density, effective heat capacity, and effective thermal conductivity of the medium, respectively. These values were then used to calculate the effective thermal diffusivity of the medium, 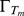, which is given by
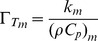
(15)The overall heat transfer parameters are highly dependent on the geometry and density of the corals. Dividing equation 11 by the 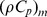 term yields a more standardised transport energy equation for porous media given as
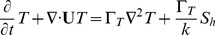
(16)The angle of incidence (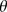) and intensity (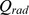) of solar radiation that strikes the coral’s surface (tissue) is also accounted for in our model. However, for the purpose of validating our models against experimental measurements, the 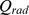 term was calculated using an overhead (90°) incident angle for solar radiation delivered to the tissue surface. Hence, equation 16can be written as
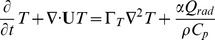
(17)


When a fluid flows over a surface, its first layer normally sticks to the boundary (no slip boundary condition). This phenomenon causes the flow to decrease, in this case, in the vicinity of the bounding coral surface, creating the momentum (velocity) boundary layer. A similar principle applies when the temperature between the fluid and surface differ. The first layer of the fluid obtains its heat from the coral surface through pure conduction. It then gives its newly acquired energy to all of the other fluid molecules through convection with which it comes in contact. This layer between the fluid and the bounding coral surface is called thermal boundary layer. In our model, equations 7 and 17directly account for these boundary layers. The ratio of the thermal vs hydrodynamic thicknesses is given by the Prandtl number
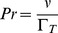
(18)


The mass, momentum, and energy conservation equations were solved over all the fluid control volumes in this computational domain using the Finite Volume Method (FVM). This solution provides results at each finite volume in the model, at discrete time intervals (for further readings on FVM, the reader is referred to [17], [32]).

### Grid Independence Analysis

Grid convergence was investigated to test whether our modelled solutions were independent of grid resolution. Our purpose here was to reduce truncation error and establish the minimum degree of grid resolution required. We performed simulations initially on coarse grids then successively finer grids to observe the effects of grid resolutions on coral surface temperatures. We varied the number of grid cells from 150,000 to 1,000,000 cells with the constant time step of 1.0 s. As the grid becomes finer, the solution should become less sensitive to grid spacing and approach an asymptotic convergence value. We tested a range of coral species with massive and branching growth morphologies for grid convergence (details are below).

### Model Validation

We used the experimental study of Jimenez *et al.* [8] to validate our CFD models. Jimenez *et al.* conducted their experiments in a flow chamber (10 cm height × 5 cm width × 25 cm length) with a sand covered floor. A steady inlet flow was maintained in the horizontal (length) direction. Measurements of coral surface temperatures of cylindrical branches cut from corals were made in their flow chamber [8]. They explored coral surface temperatures under two environmental temperature scenarios: steady-state and transient.

In their steady-state experiment, flow was maintained at 0.002 m s^−1^, and temperature at 26°C. Heat was provided by a fiber-optic light source with a collimating lens to focus light on the coral surface. The same experimental setup was also used outdoors, where corals (*Porites lobata* and *Stylophora pistillata*) were exposed to direct sunlight ranging from approximately 500 to 950 W m^−2^. Coral surface warming was estimated as the difference between coral surface temperature and ambient water temperature after 30 min of exposure to direct solar irradiance. Downwelling irradiance was measured with a submersible light logger, while temperature measurements of both water and coral surface were made using thermocouples connected to a data logger.

The transient experiments, in contrast, were done to monitor the effects of temperature changes at the surface of corals under changing light regimes. The corals (*Cyphastrea serailia* and *Seriatopora hystrix*) were heated by direct illumination using a fiber optic light source (spectral range from 400–730 nm) fitted with an adjustable lens. Corals were held in darkness for approximately 5 min before a dark-light shift was imposed, employing an irradiance of 600 W m^−2^. Temperatures were estimated by inserting microsensors into the coral tissue or into the mouth of a polyp. The point source temperatures were then averaged for each coral, in order to obtain an average tissue (surface) temperature.

Another set of steady-state experiments were carried out using two hemispherical colonies (*P. lobata* and *Favia* sp.) and one branch of *S. pistillata* to investigate thermal boundary layer properties. Here temperature profiles were measured at two irradiances (1,080 and 2,080 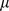 mol photons m^−2^ s^−1^; approximately 310 and 600 W m^−2^) using two flow rates (0.002 m s^−1^ and 0.013 m s^−1^). Based on their empirical models, Jimenez *et al.* suggested the light absorptivity of the tissue of the three coral species used in their steady-state experiment ranged from 0.13 to 0.28, depending upon species and pigmentation levels of individual colonies.

### Model Configurations

**Figure 2:**
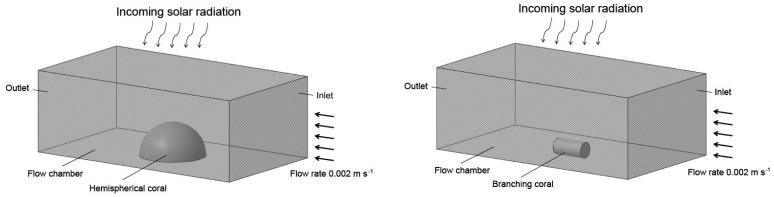
Conceptual representation for both models and the experiment apparatus used by Jimenez *et al.* * Left:* massive/hemispherical coral. *Right:* branching coral.

To simulate the conditions used by Jimenez *et al.*, three-dimensional geometries for massive and branching corals were developed (Figure 2 (a) and (b), respectively). The low Reynolds numbers estimated for their experimental setup indicated laminar flow conditions (Table 2). Coral geometries were modeled for both thermal regions, tissue and skeleton, each with its own thermal performance functions. The diameters and lengths of branching corals modeled here are the same as those used by Jimenez *et al.* [8].

**Table 2:** Dimensions of corals used by Jimenez *et al.*

Species Name	Used in Experiment	Diameter(mm)	*Re*
*Porites lobata*	Steady State	35 (hemisphere)	72^a^ & 468*^b^*
*Stylophora pistillata*	Steady State	6 (cylindrical, 6 mm long)	12^a^ & 80*^b^*
*Favia sp.*	Steady State	35 (hemisphere)	72^a^ & 468*^b^*
*Cyphastrea serailia*	Transient State	50 (hemisphere)	100^a^
*Seriatopora hystrix*	Transient State	3 (cylindrical, 6 mm long)	6^a^

a indicates flow of 0.002 m s^−1^, *^b^* indicates flow of 0.013 m s^−1^.

### Skeletal Bulk Density Measurements

The mean bulk and true densities and porosity were estimated for cores of coral skeletons to provide data for modelling flow permeability through them. Representative samples of each species used by Jimenez *et al.* were assessed (except for the hemispherical coral *C. serailia*, which was unavailable). Skeletal samples were sectioned into defined volumes, dried in an oven at 105°C, then weighed to estimate the skeletal bulk density (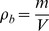). Porosity values were then calculated using equation 19. Porosity 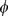 is defined as the fraction of the total volume of a medium, which is occupied by void space. Thus 1 - 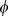 is the fraction that is occupied by solid material.
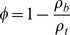
(19)where *p_b_* is the bulk density, and *p_t_* is the true density. The estimated (and assumed) porosity values for each of the species examined by Jimenez *et al.* are presented in Table 3.

**Table 3:** Estimated skeletal porosities.

Species Name	Skeletal Porosity
*Porites lobata*	0.475±0.003
*Stylophora pistillata*	0.431±0.003
*Cyphastrea serailia*	0.500*
*Seriatopora hystrix*	0.405±0.003

* Species not available. Realistic value for massive coral assumed.

### Sensitivity Analysis

Our estimates of skeletal bulk density of each coral species may have been prone to experimental error. If so, such error could have implications for predicting coral skeleton temperatures. To examine the potential accuracy of our skeletal bulk-density predictions to the rate of temperature rise for hemispherical and branching coral skeletons, the volumes of each coral sample were varied ± 10% at a constant heat flux of 750 W/m^2^ and steady inlet flow of 0.002 m s^−1^. Table 4 summarises the parameters used for sensitivity analysis on hemispherical and branching coral skeletons.

**Table 4:** Parameters used in the sensitivity analysis.

Inlet reference pressure	0 Pa
Inlet temperature	26°C
Inlet velocity	0.002 m/s
Skeletal bulk volume	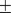 10%
Heat flux	750 W/m^2^

### Numerical Simulations

In order to adequately simulate the flow conditions corresponding to the experimental work of Jimenez *et al.*, it was necessary to assign initial and boundary conditions to the flow domain. Fixed-value boundary conditions for superficial velocities of 0.002 m s^−1^ and 0.001 m s^−1^ were applied at the inlet and bottom domains, respectively. A zero-gradient boundary condition constrains the normal gradient of the boundary patch to zero, while the slip boundary condition sets the normal velocity component to zero. The pressure at the inlet was fixed at the zero gradient condition. The pressure at the outlet were fixed at the reference pressure, while the outlet velocity was fixed at the zero gradient. The velocity and pressure at the domain sides was fixed at the slip boundary condition. The initial and boundary conditions for velocity, pressure, and temperature are shown in Table 5.

**Table 5:** Initial and boundary conditions for models.

Parameter	Initial Value	Walls	Top	Bottom	Inlet	Outlet
reference pressure (*p*)*_ref_*	0 Pa	S	S	ZG	ZG	FV
velocity (*u*)	0.002 m s^−1^	S	S	FV 0.001 m s^−1^	FV	ZNG
temperature (*T*)	26°C	S	S	FV	FV	ZG

S: Slip, ZG: Zero Gradient, ZNG: Zero Normal Gradient, FV: Fixed Value.

Our transient models used the Euler time scheme for solving ordinary differential equations. The mesh near the coral was finer than anywhere else in the domain in order to adequately capture the momentum and thermal boundary layers. We used two types of grids, predefined block structured hexahedral and polyhedral, to represent both computational and coral domains with approximately 900,000 cells each depending on the shapes and sizes of the corals being considered. To decrease simulation time, computations were run in parallel using 4 CPU’s using decomposition methods in OpenFOAM.

We used the SIMPLE (semi implicit method for pressure-linked equations) and PIMPLE (merged PISO-SIMPLE) algorithms for our steady-state and transient simulations, respectively [30]. The SIMPLE algorithm determines pressure on a staggered grid from velocity components by applying an iterative procedure coupled with the Navier-Stokes equations, whereas the PIMPLE combines SIMPLE which solves the pressure-velocity coupling implicitly while the PISO (pressure implicit with splitting of operators) algorithm rectifies the second pressure correction and corrects both velocities and pressures explicitly [17], [30], [32].

## Results

### Grid Independence Analysis

**Figure 3:**
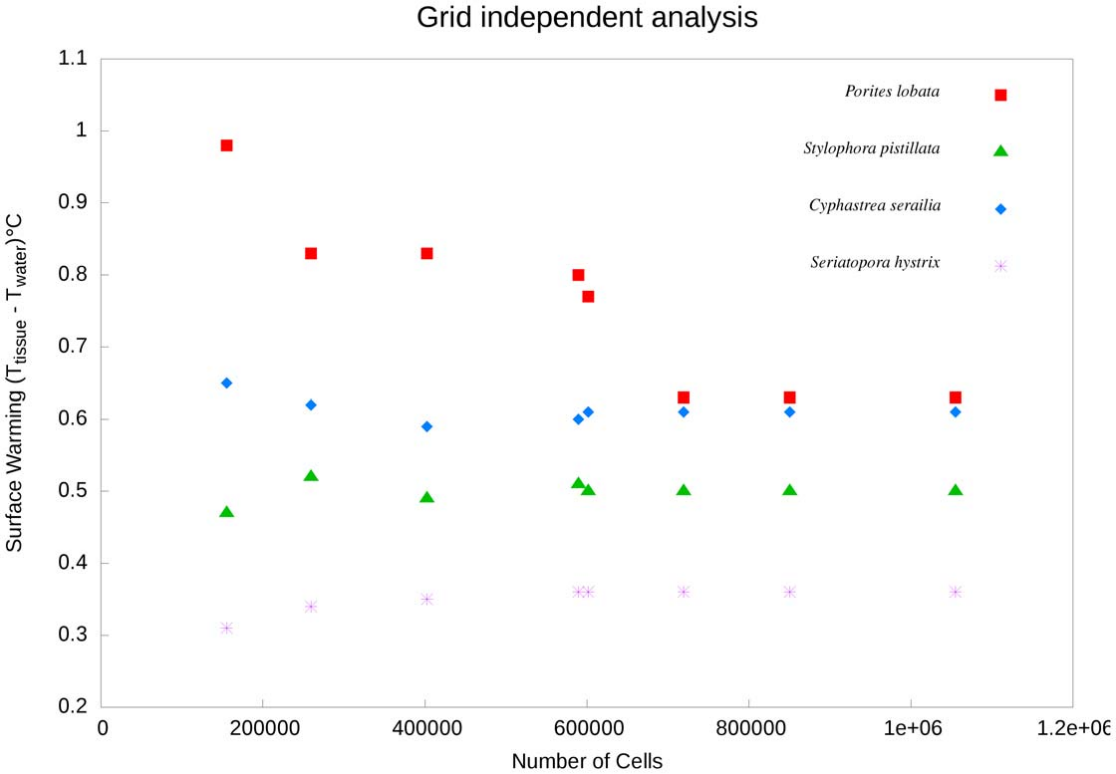
Grid independence analysis based on varying the number of grid cells at constant flow (0.002 m s**^−1^**) and two irradiances (600 W m**^−2^** for ***C. serailia*** and ***S. hystrix*** and 750 W m**^−2^** **** for ***P. lobata*** and ***S. pistillata***).

Except for *P. lobata*, the number of cells needed to reach steady-state coral tissue warming was approximately 600,000. For *P. lobata*, the surface warming oscillated strongly and only converged on a solution at approximately 800,000 cells (Figure 3). These results suggest that the grids used in our models (900,000 cells) were sufficiently fine to capture these heating dynamics, and therefore, our solutions were derived within this asymptotic range. Further refinement of the grid would be unlikely to yield significant variations to our results.

### Steady State Simulation

**Figure 4:**
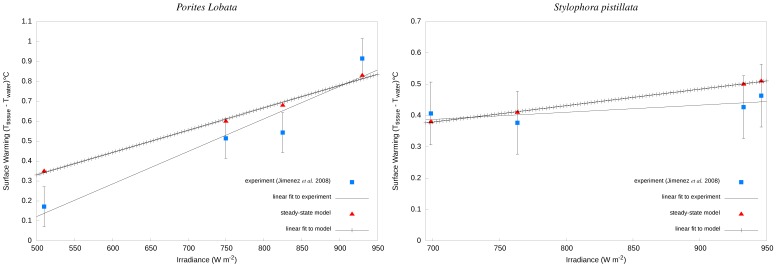
Comparisons of simulated and experimental surface warming of corals. Coral surface warming is given as a deviation from the ambient water temperature (26°C) under a constant inflow condition (0.002 m s^−1^) and direct solar irradiances. *Left:* Surface warming of hemispherical *P. lobata*. The linear correlation coefficients (R^2^) for the experimental and modelled datasets were 0.92 and 0.99, respectively. *Right:* Surface warming of a branch of *S. pistillata*. The linear correlation coefficients (R^2^) for the experimental and modelled datasets were 0.59 and 0.99, respectively.

For the hemispherical coral, *P. lobata*, the temperature differential increased with increasing irradiance at a flow rate of 0.002 m s^−1^ (Figure 4). However, for the branching coral, *S. pistillata*, the surface warming showed no significant increase with increased irradiance at the same flow rate (Fig. 4). For *S. pistillata*, the branching coral, the *A*/*V* ratio was 1,666, and for *P. lobata*, the hemispherical coral, the *A*/*V* ratio was 257.

Both the experimental data of Jimenez *et al.* and the CFD models agreed well with a linear relationship between coral surface warming and irradiance; consistent with heat transfer theory. The correlation coefficient between the modelled results and a straight line fit was R^2^ = 0.99 for both branching and hemispherical corals. Whereas for the experimental results, R^2^ values of 0.59 and 0.92 were calculated for branching and hemispherical corals against linear fits to each data set, respectively. The greatest temperature difference between the experimental and simulated data was 0.15°C (22%) for the hemispherical coral at 825 W m^−2^. The average temperature difference between the models and experimental results for branching coral and hemispherical coral were 0.03°C and 0.13°C, respectively. An increase in flow velocity from 0.002 m s^−1^ to 0.013 m s^−1^ under constant irradiance of 600 W m^−2^ resulted in a slight decrease of surface warming for all coral species (Table 6).

**Table 6:** Effect of low flow (0.002 m s^−1^) and high flow (0.013 m s^−1^) on coral surface warming under constant irradiance of 600 W m^−2^.

	*Favia sp.*		*S. pistillata*		*P. lobata*	
Parameters	Low flow	High flow	Low flow	High flow	Low flow	High flow
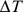 (experiment)	0.56±0.01	0.35±0.01	0.38±0.03	0.16±0.01	0.60±0.14	0.57±0.01
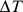 (model)	0.50	0.35	0.35	0.21	0.58	0.52

### Transient State Simulation

**Figure 5:**
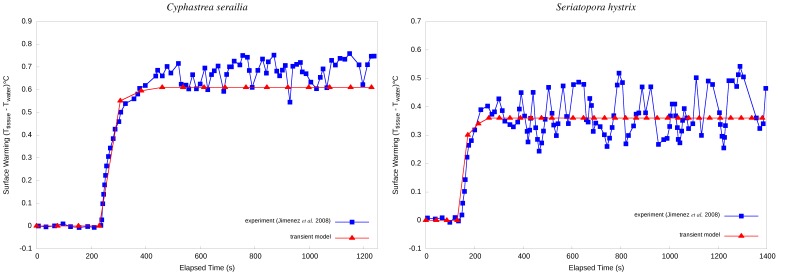
Surface warming of corals in response to a dark-light shift. *Left:* hemispherical *C. serailia*. *Right:* branch of *S. hystrix.*

Dark-light shift simulations (see the model validation section) were performed for the massive coral *C. serailia* and the branching coral *S. hystrix*. Illumination commenced at 143 s and 235 s for hemispherical and branching corals, respectively (Figure 5). The time constants from both the modelled and experimental data after the onset of illumination were nearly twice as long for the hemispherical coral (340±10 s and 310±10 s, respectively) than for the branching coral (180±10 s and 220±10 s, respectively). The calculated *A*/*V* ratios of the hemispherical coral (*C. serailia*) and the branching coral (*S. hystrix*) were 180 and 3,000, respectively. These differences in *A*/*V* ratios were consistent with the differences in time constants.

The results of our transient-state simulations agreed well with the experimental results of Jimenez *et al.* [8], as well. The smaller temperature increase experienced by the branching coral compared to the hemispherical coral was also consistent with theoretical expectations and their experimental results. Mean temperature differences between their experiments and our simulations in hemispherical and branching corals were 0.08°C and 0.03°C, respectively.

### Flow Patterns Inside Corals

**Figure 6:**
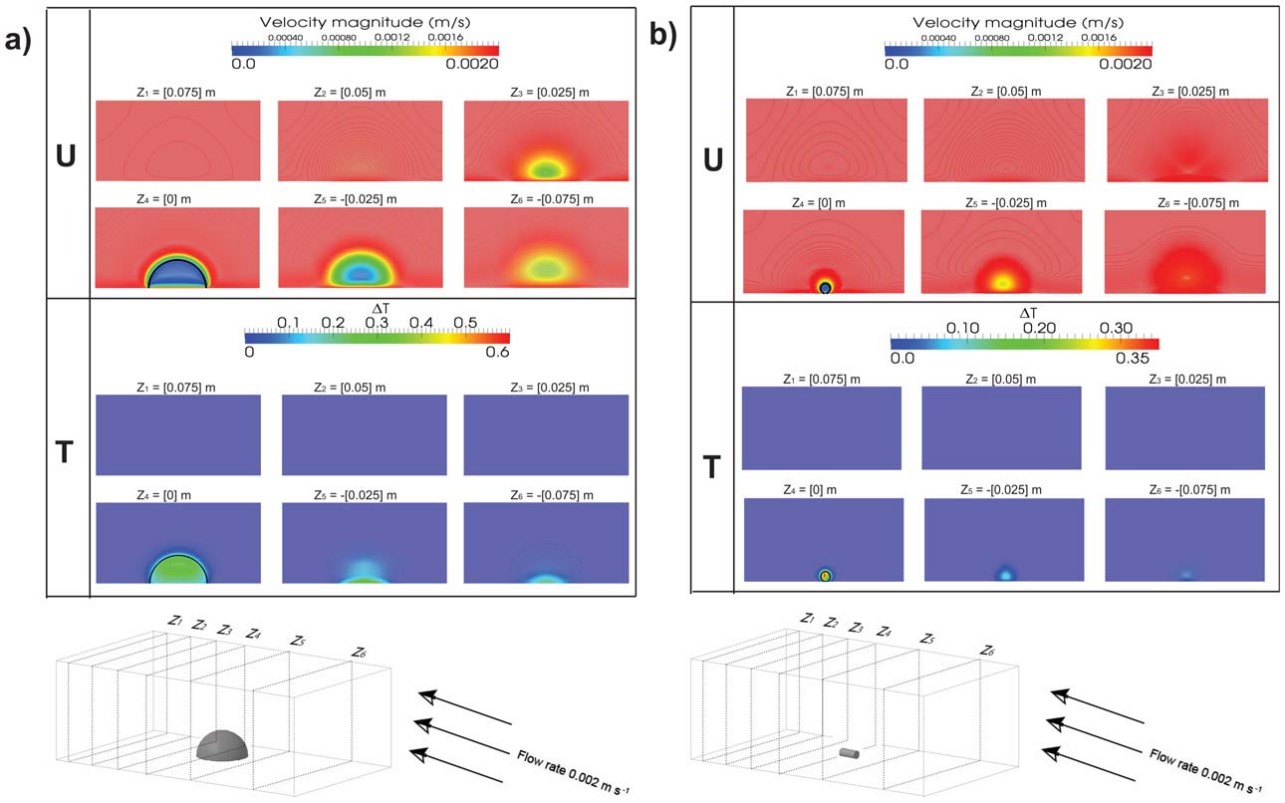
Slices of axial velocity, temperature magnitude, and contour for six different cross sectional planes. The inlet boundary condition is a constant velocity of 0.002 m s^−1^. At Z = 0 [m] is the middle slice through the center of the coral, perpendicular to flow. Z<0 is in the upstream direction. The coral cross section is shown as a thick black line in the 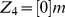 plane. *a):* Fields of axial velocity and temperature in hemispherical *P. lobata*. *b):* Fields of axial velocity and temperature in a branch of *S. pistillata.*

The two-dimensional velocity magnitude and coral surface warming at different cross-sectional planes are shown in Figure 6 for both hemispherical and branching corals. Note that only one slice has coral within its bounds, the coral cross section is shown as a thick black line in the 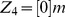 plane. Due to the absence of turbulence, the flow fields show homogenous distributions throughout the domain and isotropic behaviour of coral tissue and skeleton.

### Sensitivity Analysis

**Figure 7:**
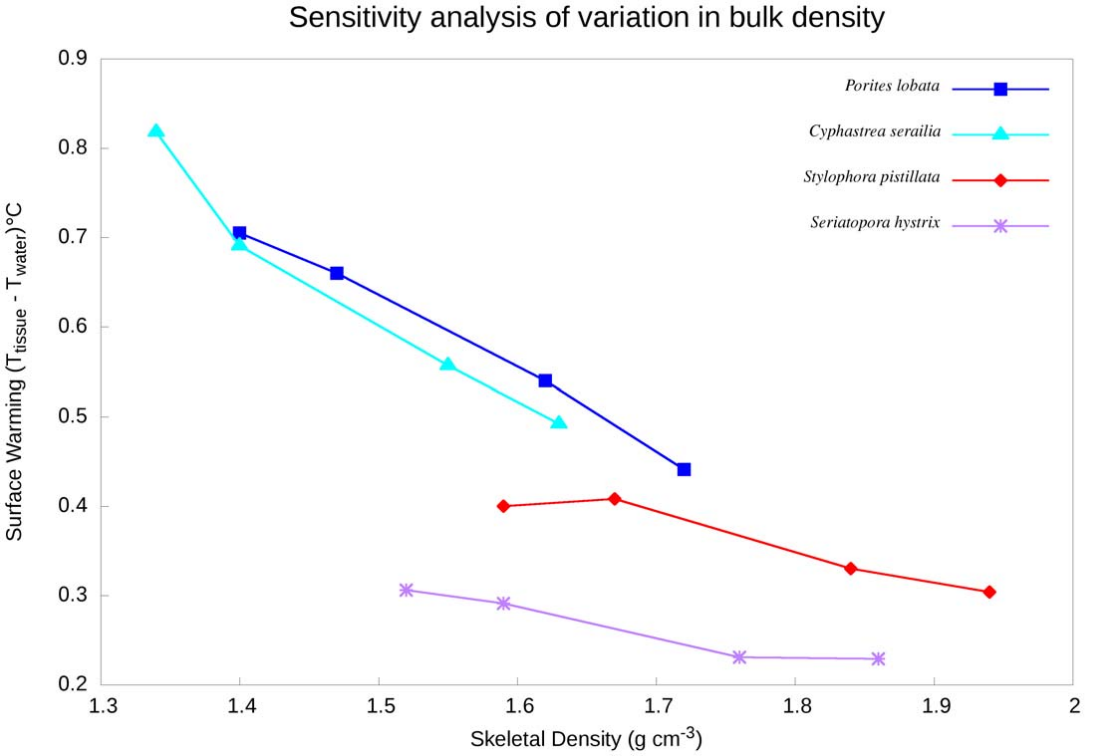
Sensitivity analysis of skeletal temperature rise to variations in bulk density under constant irradiance of 750 W m**^−2^**.

An increase in bulk density at a constant heat flux decreased the overall surface temperature for hemispherical coral in a similar but inverse manner to porosity changes (Figure 7). However, the bulk density variation for branching coral had little or no effect on overall temperature rise.

## Discussion

The study of thermal microenvironments of corals in response to environmental variables can enhance our understanding of the mechanisms responsible for coral bleaching. The thermal microenvironment can also significantly affect many other temperature controlled processes, such as coral metabolism and growth [7]. Therefore, the CFD models and techniques presented here have the potential to facilitate a better understanding of how irradiance and flow are related to patterns of coral bleaching and how this might vary among coral species, individual corals, and locations of individual coral colonies on the reef.

Our simulation results were broadly consistent with the experimental results of Jimenez *et al.* with respect to thermal and temporal responses of corals under irradiation, and the effects of coral shape and size. In steady-state light conditions, the effects of *A*/*V* ratios was as expected given heat loss should increase with increasing surface area and decrease with decreasing volume. The higher *A*/*V* ratio of the branching coral *S. pistillata* compared to the hemispherical coral *P. lobata* was consistent with the expectation from theory that heat loss should be greater with a branching morphology. Under transient light conditions, the higher *A*/*V* ratio of the branching coral *S. hystrix* means that heat would be dissipated to the environment faster than it would be from the hemispherical/massive coral, *C. serailia*. Therefore, under equivalent illumination a longer time would be required for branching corals of an equivalent volume/mass to reach equilibrium with their immediate thermal environment. Typically, however, massive corals have a considerably greater volume/mass ratio, and as we observed here, hemispherical corals heated at a slower rate, taking nearly twice as long to equilibrate. In this case, the effect of faster cooling resulting from different *A*/*V* ratios was over taken by thermal mass effects. Temperature values were obtained for each finite volume over the entire coral surface area in our CFD simulations, which contrasted with the microsensor approach of the experimental study [8]. In this way, the highest temperature reached on a coral’s surface (for our idealised geometry) was easily obtained.

An increase in skeletal bulk density (inversely proportional to porosity) may result in an increase in the viscous and inertial resistance where forced convection becomes ineffective. The penetration of flow through the coral skeleton will reduce the heat transfer coefficient and lead to decreased fluid residence time and contact surface area between coral skeleton and fluid. As a result, the intensity of energy transfer between phases will reduce the temperature gradient of the coral skeleton. Given substantial inter-colony variation in skeletal bulk density and porosity [10], such physical features of coral may be useful for predicting coral bleaching responses. It should, however, be noted that flow through corals would normally only be expected in injured (damaged/predated) corals, which would therefore already be stressed.

### Future Directions

While our work here focused on laboratory scale models, we also recognise the potential benefits of simulations of more realistic environments. The physics of in-situ field simulations, though, poses many challenges. Among these challenges are how best to model turbulence, variation in solar irradiance and atmospheric conditions for any given geographic position and time of day, and estimation of subsurface flow magnitude for any given depth. Some opportunities to address the issues, however, do exist. For example, turbulence could be modelled using the Reynolds Averaged N-S (RANS) model. These models provide good representation of turbulence, with reasonably low computational demands. Temporal and spatial variation in irradiance and spectral quality, cloud cover, water depth and turbidity, and sea-state could be accommodated using a ray-tracing algorithm combined with tidal and climatic data. Subsurface flow, on the other hand, could be handled by using three dimensional hydrodynamic models [33].

A more complete study of the influence of these parameters on bleaching, is the utlimate aim of our work. Such a study however, is beyond the scope of this paper. Instead, this paper is intended to introduce the possibility of understanding coral bleaching from the perspective of heat transfer in a CFD framework.

### Concluding Remarks

Here we have introduced CFD models as a powerful new tool for investigating the thermal microenvironment of corals. In this work, numerical computations were developed using the open-source, C++ library platform, OpenFOAM. The simulations implemented laminar flow conditions and a first order discretisation scheme, in order to simulate the initial and boundary conditions of the experimental work of Jimenez *et al.* [8]. Our CFD models, however, are sufficiently flexible and capable of accommodating the physics of large-scale in-situ modelling. Using the CFD techniques presented here, we were able to investigate parameters of interest inside and at the surface of corals [15], [16], [34]. Doing so would be difficult, if not impossible, by other means. In combination with more sophisticated and detailed CFD solvers, Computed Tomography (CT) imaging of corals may allow us to develop even finer-scale models. Doing so may subsequently enhance our understanding of these phenomena further. From our initial work in this area, it would seem that CFD is a valuable tool for understanding coral bleaching and/or thermal responses of corals, and therefore, has the potential to be applied more widely to the issue of coral bleaching in the future.
